# Interaction of dendritic cells and T lymphocytes for the therapeutic effect of *Dangguiliuhuang* decoction to autoimmune diabetes

**DOI:** 10.1038/srep13982

**Published:** 2015-09-11

**Authors:** Tingting Liu, Hui Cao, Yachun Ji, Yufeng Pei, Zhihong Yu, Yihong Quan, Ming Xiang

**Affiliations:** 1 Department of Pharmacology, School of Pharmacy, Tongji Medical College, Huazhong University of Science and Technology, Wuhan, 430030, China; 2 The Central Hospital of Wuhan, Wuhan 430014, China

## Abstract

In traditional Chinese medicine (TCM), *Dangguiliuhuang* decoction (DGLHD) is an effective treatment of autoimmune diabetes. Here, we studied potential anti-diabetic mechanisms of DGLHD in a non-obese diabetic (NOD) mouse model. *In vitro,* DGLHD and individual active ingredients enhanced glucose uptake in HepG2 cells, inhibited T lymphocyte proliferation, and suppressed dendritic cells (DCs) function. *In vivo*, DGLHD significantly inhibited insulitis, delayed the onset and development of diabetes, promoted insulin secretion and sensitivity, and balanced partially normalized Th1 and Th2 cytokines in NOD mice. In addition, DGLHD increased α_1_-antitrypsin (AAT-1), Bcl-2, and CyclinD1, and decreased Bax levels in pancreas, spleen, thymus, DCs, and a NIT-1 cell line, all consistent with protecting and repairing islet β cell. More detailed studies indicated that DGLHD regulated the maturation and function of DCs, decreased the percentage of merocytic dendritic cells (mcDCs) subset, and increased programmed death ligand-1 (PD-L1) expression in DCs. DGLHD also impeded T lymphocyte proliferation and promoted regulatory T cells (T_regs_) differentiation *in vivo*. A JAK2-STAT3-dependent pathway was involved in the suppression by DGLHD of interactions between DCs and T lymphocyte. The experiments implicated five active ingredients in specific anti-diabetic actions of DGLHD. The results demonstrated the reasonable composition of the formula.

Type 1 diabetes mellitus (T1DM) is an autoimmune disorder caused by the selective destruction of insulin-producing β cell^1^. The development of T1DM involves complex interactions between pancreas islet β cell and immune cells, including dendritic cells (DCs), auto-reactive T cells, and regulatory T cells (T_regs_)^2^. DCs are the most powerful antigen-presenting cells (APCs) and are central to the autoimmune process in T1DM^3^. DCs can induce the conversion of self-reactive CD4^+^ T cells into T_regs_ and promote immunotolerance in non-obese diabetic (NOD) mice^4^. Although T-cell abnormalities are important components of autoimmune disorders^5^, the mechanisms of adverse interactions between DCs and T-cell in T1DM remain unclear.

Disorders of immune homeostasis induce a complex network of intracellular signaling cascades to induce T1DM^6^, including the Janus kinase (JAK)/signal transducer and activator of transcription (STAT) pathways. In animal models, neutralization of JAK2-STAT3 signaling appears to protect against T1DM^7^. Recently, TLR4 has also been shown to contribute to the pro-inflammatory state and autoimmune destruction of islet β cell during the development of T1DM^8^.

In traditional Chinese medicine (TCM), combinations of plant species and minerals, called formulae, are often prescribed based on clinical experience. Nearly 100,000 formulae have been recorded, but for most the mechanisms of action remain unknown^9^. Typically, formulae consist of several types of medicinal herbs or minerals, in which one represents the principal component, and others serve as adjuvants contributing to the effects or to facilitate the delivery of the principal component. In some formulae, multiple components could interact with multiple targets and exert synergistic therapeutic efficacies^10^. *Dangguiliuhuang* decoction (DGLHD) is a compound Chinese medicine recorded in “*Lan Shi Mi Cang*” which has positive effects on diabetes and its complications. DGLHD formula consists of principal components (*angelica sinensis*, *radix rehmanniae,* and *radix rehmanniae preparata*) and adjuvant components (*radix scutellariae*, *rhizoma coptidis*, *cortex phellodendri,* and *radix astragali*). The seven herbs are decocted in distilled water at the ratio of 1:1:1:1:1:1:2. The major active ingredients of DGLHD are principal components (ferulic acid and catalpol), adjuvant components (baicalin, berberine, and astragaloside IV). Multiple clinical reports indicate that DGLHD and its components improve the complications and blood glucose fluctuations in diabetic patients (Table 1)^11^.

Here, we investigated possible antidiabetic mechanisms of DGLHD and its five active ingredients *in vitro* and in a NOD mouse model of T1DM *in vivo*. The results reveal specific cellular targets and signal pathways involved in the marked antidiabetic actions of DGLHD.

## Results

### DGLHD treatment delayed the onset and development of diabetes

**Figure 1:**
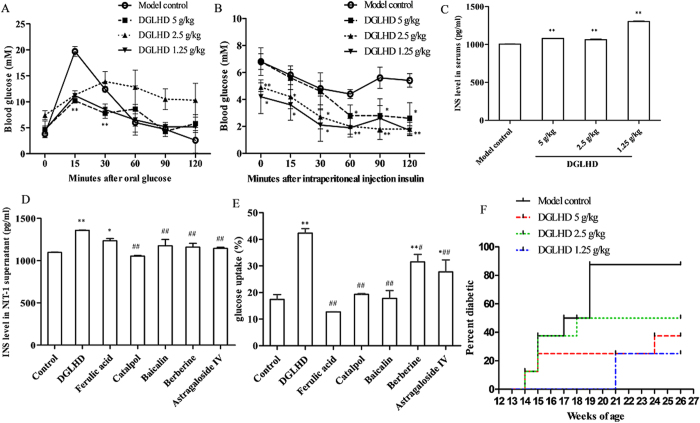
DGLHD treatment delayed the onset and development of diabetes. (**A**) OGTT of NOD mice treated with DGLHD for 4 weeks from 8 until 12 weeks of age or model control animals (*n *= 6). (**B**) ITT of NOD mice treated with DGLHD or model control animals (*n *= 6). (**C**) Effect of DGLHD on the serum insulin secretion (*n *= 3). (**D**) Effect of DGLHD and ingredients on the insulin secretion in NIT-1 supernatant (*n *= 3). (**E**) Effect of DGLHD and the active ingredients on the glucose uptake of HepG2 (*n *= 3). (**F**) Diabetes incidence in NOD mice at 26 weeks of age treated with or without DGLHD (*n *= 8). Data were presented as means ± SD. ^*^P < 0.05, ^**^P < 0.01 compared with model control/control group. ^#^*P *< 0.05, ^##^*P *< 0.01 compared with DGLHD group.

At 12 weeks of age, NOD mice treated with DGLHD were more insulin-sensitive than the model controls as quantified by oral glucose tolerance tests (OGTT) and insulin tolerance tests (ITT) (Fig. 1A,B). DGLHD increased insulin secretion in both NOD mice (Fig. 1C) and NIT-1 cell (Fig. 1D). Ferulic acid, one of the principal components of DGLHD, also increased insulin secretion, but was less efficacious than DGLHD. The effects of other active ingredients were not remarkable (Fig. 1D). DGLHD and the active ingredients berberine and astragaloside IV increased HepG2 glucose uptake significantly. The action of DGLHD was more robust than any of the five ingredients individually (Fig. 1E). By 26 weeks of age, DGLHD treatment resulted in fewer and less severely diabetic mice than the controls treated with saline (Fig. 1F). The results indicated that DGLHD could promote insulin secretion and sensitivity in NOD, and that its effects were more attributable to the combination of multiple active ingredients rather than to any single component.

### DGLHD inhibited insulitis and increased pancreatic islet number

**Figure 2:**
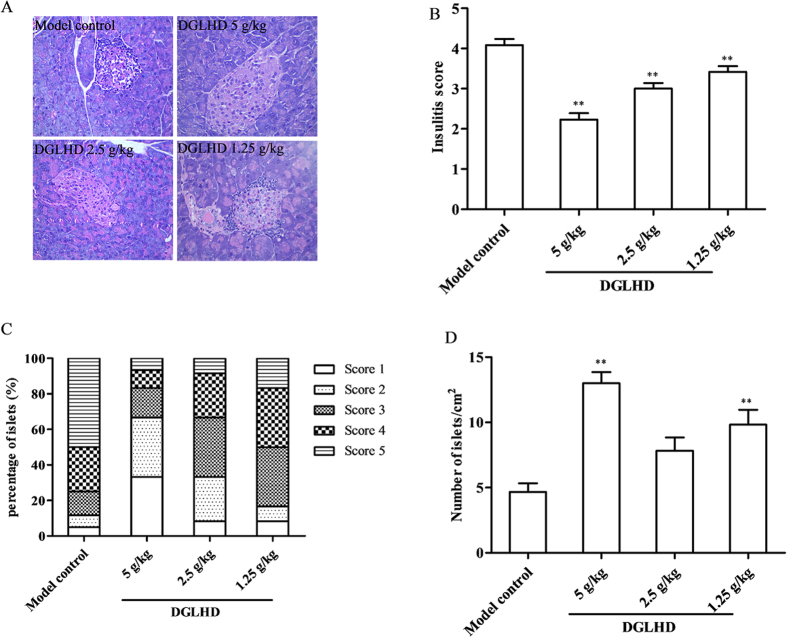
DGLHD inhibited insulitis and increased pancreatic islet number. (**A**) Representative images of pancreatic islets from 12 week-old NOD mice treated with DGLHD for 4 weeks from 8 until 12 weeks of age or model control animals. Pancreatic tissues were sectioned and stained with HE. (**B**) Insulitis score of each group. (**C**) The frequency of insulitis. (**D**) The number of islets. Data were presented as means ± SD. ^**^P < 0.01 compared with the model control group (*n *= 6).

Lymphocyte infiltration and inflammation are significant features of T1DM^12^. DGLHD protected the integrity of the islets and decreased the extent of lymphocyte infiltration (Fig. 2A). The mean insulitis score was significantly lower in DGLHD treated mice (Fig. 2B,C). In addition, the number of islets was higher in NOD mice treated with DGLHD compared to the model controls (Fig. 2D).

### DGLHD induced a Th2-biased cytokine response

**Figure 3:**
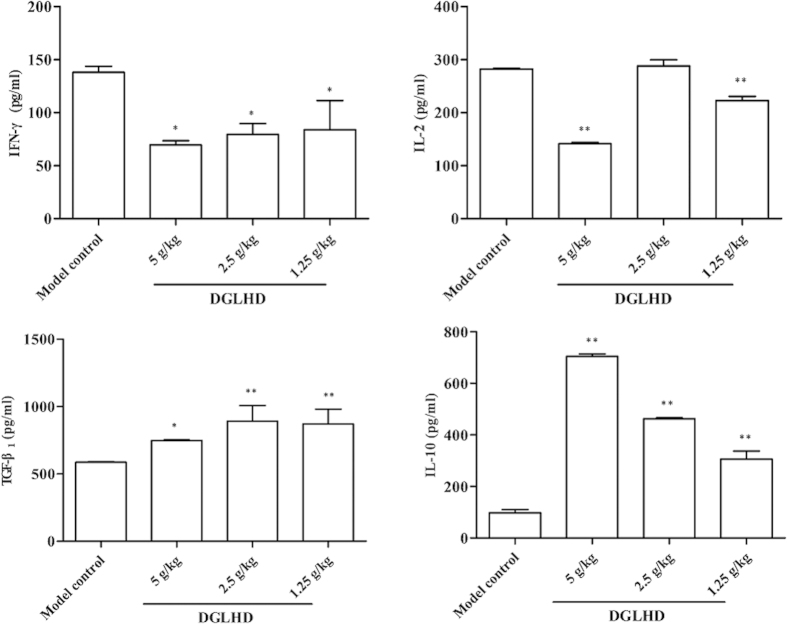
DGLHD induced a Th2-biased cytokine response. Effects of DGLHD on the production of IL-2, IL-10, IFN-γ and TGF-β_1_ in serum from 12 week-old NOD mice treated with DGLHD for 4 weeks from 8 until 12 weeks of age or model control animals. Data were presented as means ± SD. ^*^P < 0.05, ^**^P < 0.01 compared with model control group (*n *= 6).

To understand the mechanism underlying the reduction of insulitis produced by DGLHD treatment, we measured plasma autoreactive Th1/immunosuppressive Th2 cytokine levels by ELISA. Treatment of 12 week-old NOD mice with DGLHD for 4 weeks decreased production of the Th1 cytokines interferon gamma (IFN-γ) and interleukin 2 (IL-2), and increased production of the Th2 cytokines IL-10 and transforming growth factor beta one (TGF-β_1_) (Fig. 3).

### DGLHD or active ingredients functioned as pancreas islet β cells repairing

**Figure 4:**
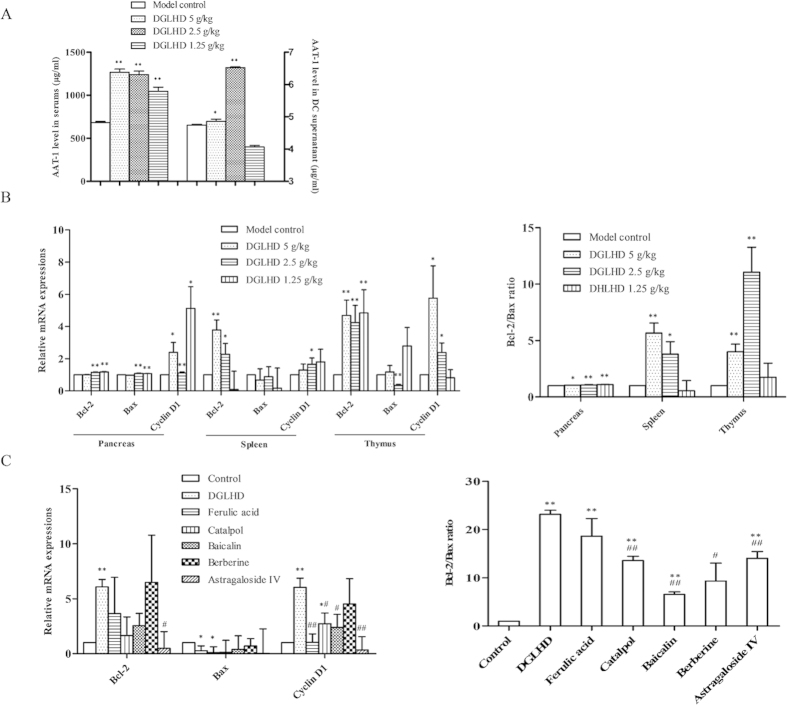
DGLHD or active ingredients functioned as pancreas islet β cells repairing. (**A**) Effect of DGLHD on the expression of AAT-1 in NOD mice serum and DCs supernatant (*n *= 6). (**B**) Effect of DGLHD on the expression of CyclinD1, Bcl-2, Bax mRNA and Bcl-2/Bax in the pancreas, spleen and thymus of NOD mice (*n *= 3). (**C**) Effect of DGLHD and active ingredients on the expression of CyclinD1, Bcl-2, Bax mRNA and Bcl-2/Bax in NIT-1 cell line (*n *= 3). Data were presented as means ± SD. ^*^P < 0.05, ^**^P < 0.01 compared with model control/control group. ^#^*P *< 0.05, ^##^*P *< 0.01 compared with DGLHD group.

Overexpression of α_1_-antitrypsin (AAT-1) protects pancreatic islet β cell from apoptosis through pathways related to caspase activity^13^. Our results here showed that DGLHD treatment of NOD mice raised the levels of AAT-1 in both serum and DCs supernatants (Fig. 4A). Treatment with DGLHD or active ingredients also increased the expressions of the anti-apoptotic gene Bcl-2 and the proliferation related gene CyclinD1, and decreased the expression of the pro-apoptosis gene Bax in pancreas, spleen, and thymus of NOD mice *in vivo* (Fig. 4B) and in the NIT-1 cell line *in vitro* (Fig. 4C). The results showed that DGLHD promoted β cell proliferation and decreased β cell apoptosis. In general, the effects of DGLHD were more pronounced than those of any of the principal or adjuvant components.

### DGLHD or individual active ingredients suppressed spleen T lymphocyte proliferation

**Figure 5:**
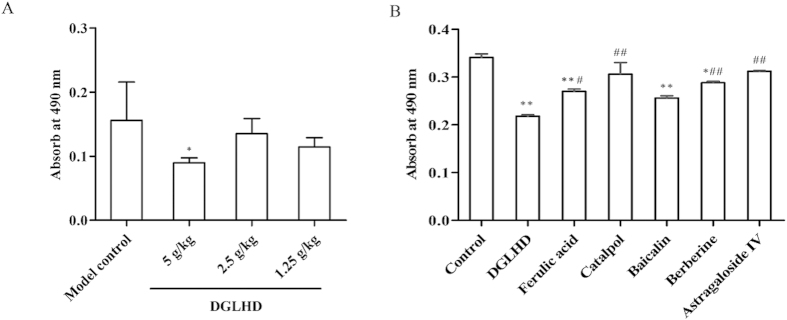
DGLHD or active ingredients suppressed T lymphocytes proliferation. (**A**) Splenic T lymphocytes were collected from 12 week-old NOD mice treated with DGLHD for 4 weeks from 8 until 12 weeks of age or model control group (*n *= 6). (**B**) Splenic T lymphocytes were collected from normal animals treated with DGLHD or active ingredients for 48 h *in vitro*. Data were presented as means ± SD (*n *= 3). ^*^P < 0.05, ^**^P < 0.01 compared with model control/control group. ^#^*P *< 0.05, ^##^*P *< 0.01 compared with DGLHD group.

Both *in vivo* and *in vitro*, DGLHD or active ingredients exhibited robust anti-proliferative effects on T lymphocytes. *In vivo*, treatment of NOD mice with DGLHD markedly suppressed spleen T lymphocyte proliferation stimulated with concanavalin A (Con A) (Fig. 5A). *In vitro*, either DGLHD or the active ingredients ferulic acid, baicalin, or berberine significantly inhibited the proliferation of normal spleen T lymphocytes (Fig. 5B).

### DGLHD enhanced CD4^+^CD25^+^Foxp3^+^ T_regs_ differentiation

**Figure 6:**
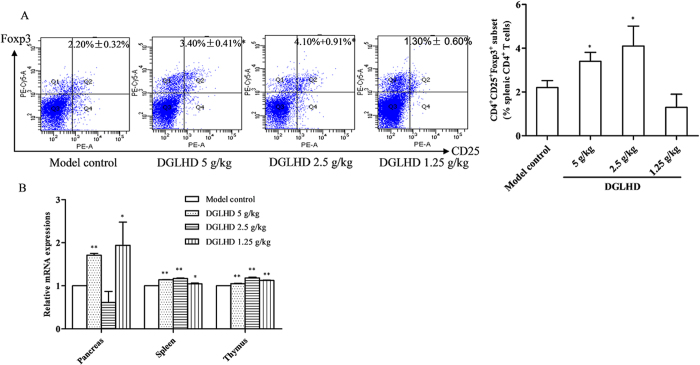
DGLHD enhanced CD4^+^CD25^+^Foxp3^+^ T_regs_ differentiation *in vivo*. (**A**) The percentage of CD4^+^CD25^+^Foxp3^+^ T_regs_ in spleen isolated from 12 week-old NOD mice treated with DGLHD for 4 weeks from 8 until 12 weeks of age or model control animals (*n *= 6). (**B**) Foxp3 mRNA expression in pancreas, spleen and thymus treated with DGLHD for 4 weeks were detected by qPCR. Data were presented as means ± SD (*n *= 3). ^*^P < 0.05, ^**^P < 0.01 compared with model control group.

CD4^+^CD25^+^Foxp3^+^ T_regs_ exhibit immune regulatory activity and protect against autoimmune diabetes development^14^. Treatment of NOD mice with DGLHD for 4 weeks increased the percentage of CD4^+^CD25^+^Foxp3^+^ T_regs_ in spleen lymphocytes (Fig. 6A). Forkhead box transcription factor (Foxp3) is essential for the differentiation and function of T_regs_, and loss of Foxp3 contributes to T1DM pathogenesis^15^. DGLHD treatment increased Foxp3 mRNA expression in pancreas, spleen, and thymus (Fig. 6B).

### DGLHD decreased maturation and increased antigen uptake of DCs

**Figure 7:**
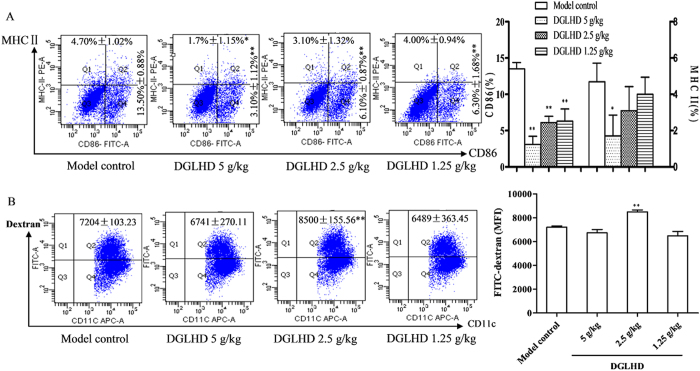
DGLHD regulated DCs maturation and antigen uptake. (**A**) Effect of DGLHD on CD86 and MHC-II expression of DCs by FACS analysis *in vivo*. Mice BMDCs were obtained from 12 week-old NOD mice treated with DGLHD for 4 weeks or the model control mice. MHC-II and CD86 were analyzed in CD11c^+^ BMDCs. (**B**) Cumulative results of FITC-dextran uptake in DGLHD treated BMDCs. Data were presented as means ± SD (*n *= 3). ^*^P < 0.05, ^**^P < 0.01 compared with model control group.

Phenotypic maturation and functional abnormalities in bone marrow DCs (BMDCs) are implicated in defective immune regulation that leads to T1DM^16^. BMDCs were generated from 12 week-old NOD mice following DGLHD treatment and cultivated with cytokine cocktails (GM-CSF and IL-4) for 7 days. More than 90% of the cultured cells were determined to be DCs, which displayed a dendritic shape. DCs are basically defined by their expression of CD11c, an established marker^17^. As expected, DGLHD treatment decreased the expressions of MHC class II (MHC-II) and co-stimulator CD86 on CD11c^+^ DCs (Fig. 7A). These results are consistent with the view that DGLHD maintained the BMDCs in an immature state and induced an immunological tolerance in NOD mice.

One of the important functions of immature DCs (iDCs) is the potential to capture and take up antigen, and this ability is down-regulated upon maturation^18^. The process of antigen capture by DCs was analyzed here using the FITC-dextran (antigen particle) uptake test. Untreated BMDCs took up less FITC-dextran than they were exposed to 2.5 g/kg DGLHD (Fig. 7B).

### DGLHD inhibited DCs migration

**Figure 8:**
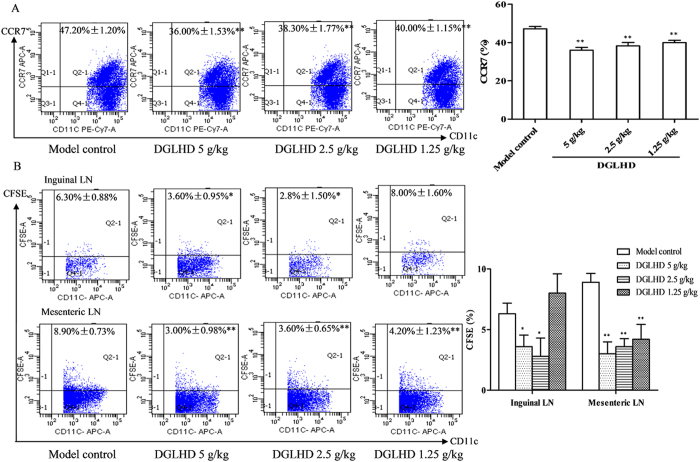
DGLHD impaired DCs migratory capacity. (**A**) The proportion of CD11c^+^CCR7^+^ DCs of NOD mice treated with DGLHD for 4 weeks from 8 until 12 weeks of age or model controls. (**B**) The proportion of CD11c^+^CFSE^+^ DCs migration to the normal mice mesenteric LN and inguinal LN. Data were presented as means ± SD (*n *= 3). ^*^P < 0.05, ^**^P < 0.01 compared with model control group.

After being activated by irradiation or chemotherapy, recipient DCs migrate from tissues to lymph nodes (LN), where they induce T cell activation. CCR7 expressed by DCs mediates the migration of activated DCs from tissues into LN^19^. We found that the proportion of CCR7 in BMDCs was reduced by DGLHD treatment (Fig. 8A). CFSE-labeled DCs, from model controls and DGLHD-treated mice, were injected to the hind paws of normal mice. Subsequent DCs migration to the mesenteric and inguinal LN was diminished after DGLHD treatment (Fig. 8B).

### DGLHD induced variations of merocytic dendritic cells (mcDCs) in bone marrow (BM), LN, spleen, and thymus

**Figure 9:**
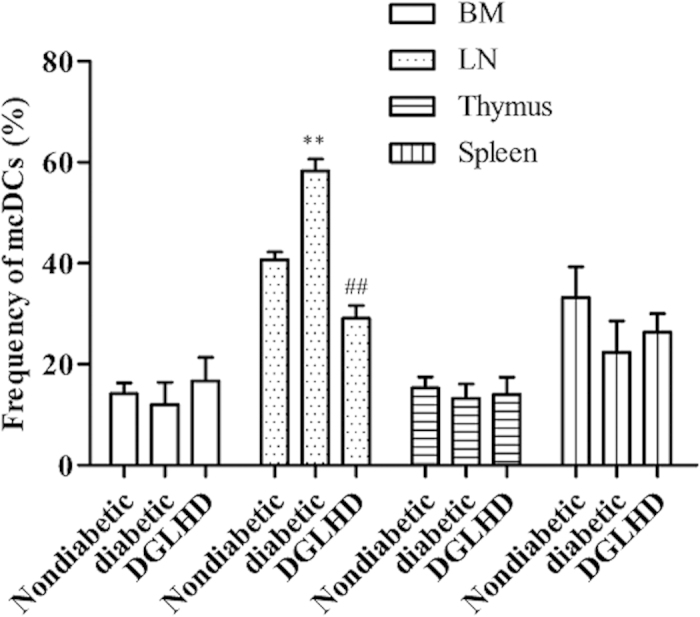
DGLHD attenuated the proportion of mcDCs in LN. DCs from non-diabetic and pathogenic NOD mice, with or without DGLHD treatment, were examined by CD11c^+^CD11b^−^CD8a^−^PDCA^−^ gated cells. Data were presented as means ± SD (*n *= 3). ^**^P < 0.01 compared with non-diabetic group. ^##^*P *< 0.01 compared with diabetic group.

CD11c^+^CD11b^−^CD8a^−^PDCA^−^ mcDCs is an unconventional DCs subset which can reverse T cell tolerance. The effectiveness of mcDCs in diminishing T cell tolerance has been demonstrated in the context of both tumorigenesis and autoimmunity^20^. Thus, modulating the number of mcDCs *in vivo* during the development of T1DM may be of clinical interest. In this study, the number of mcDCs was elevated in BM, LN, spleen and thymus of diabetic mice. DGLHD treatment decreased the numbers of LN mcDCs toward the lower levels in the nondiabetic controls. There were no significant differences in numbers of mcDCs among the three groups in spleen, BM or thymus (Fig. 9).

### DGLHD or active ingredients regulated the interaction of DCs and T lymphocyte

**Figure 10:**
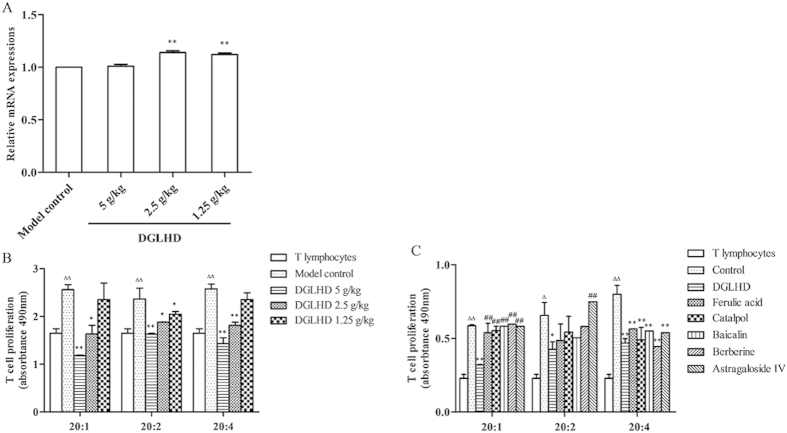
DGLHD induced PD-L1 expression and reduced the ability of DCs to stimulate T lymphocytes proliferation. (**A**) The PD-L1 mRNA expression in BMDCs. BMDCs came from 12 week-old NOD mice treated with DGLHD for 4 weeks from 8 until 12 weeks of age or model control animals (*n *= 3). (**B**) DGLHD down-regulated allogeneic MLR *in vivo*. DCs generated from NOD mice treated with or without DGLHD (*n *= 3). (**C**) DGLHD or active ingredients down-regulated allogeneic MLR *in vitro*. DCs generated from NOD mice treated with DGLHD or active ingredients for 48 h (*n *= 3). Data were presented as means ± SD. ^*^P < 0.05, ^**^P  <0.01 compared with the model control/control group. ^##^*P *< 0.01 compared with DGLHD group. ^Δ^*P *< 0.05, ^ΔΔ^*P *< 0.01 compared with the T lymphocytes without DCs stimulation.

PD-L1, also known as B7-H1, expressed in DCs. PD-L1 is mainly described as a negative regulatory molecule, and it has been frequently demonstrated that the expression of PD-L1 in DCs is correlated with the ability of DCs to induce tolerance^21^. We assessed the expression of PD-L1 in DCs with qPCR. The results showed that PD-L1 expression in DCs increased in mice treated with DGLHD for 4 weeks (Fig. 10A).

We also investigated the function of BMDCs on T lymphocyte proliferation by mixed lymphocyte reaction (MLR) ex vivo. BMDCs were generated from 12 week-old NOD mice following DGLHD treatment and then co-cultured with allogeneic T lymphocytes. The results indicated that DCs treated with DGLHD or active ingredients inhibited the ability of BMDCs to stimulate T lymphocyte proliferation. Overall, the effect of DGLHD was more pronounced than that of the active ingredients individually (Fig. 10B,C). DGLHD thus suppresses DCs maturation and function and attenuates T-cell-mediated inflammation immunity as it improves the diabetic state in NOD mice.

### DGLHD blocked JAK2-STAT3 signaling pathways, but not TLR4-mediated pathway

**Figure 11:**
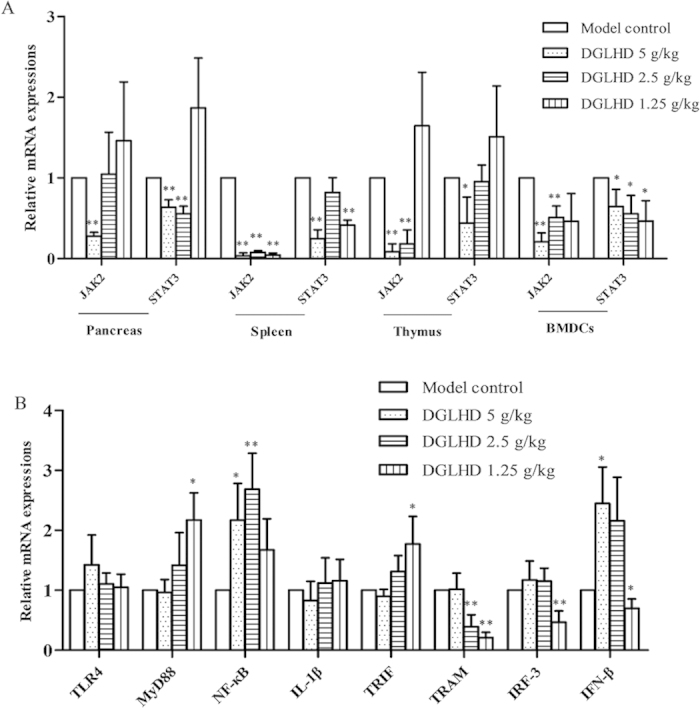
DGLHD blocked JAK2-STAT3 signaling pathways, but not TLR4-mediated signaling pathway. (**A**) DGLHD reduced JAK2 and STAT3 genes expression in pancreas, spleen, thymus and BMDCs. (**B**) The mRNA expression of the key molecules in TLR4-mediated MyD88-dependent pathway and TRIF-dependent pathway was not down-regulated by DGLHD as a whole in the pancreas. The samples come from 12 week-old NOD mice treated with DGLHD for 4 weeks from 8 until 12 weeks of age or model control animals. Data were presented as means ± SD (*n *= 3). ^*^P < 0.05, ^**^P < 0.01 compared with the model control group.

The JAK2-STAT3 pathway is an important mediator of cellular inflammatory responses in T1DM. Our results showed that DGLHD decreased JAK2 and STAT3 mRNA expression in pancreas, spleen, thymus, and BMDCs of NOD mice (Fig. 11A). We also studied mRNA expression of the key molecules in two additional pathways: 1) the TLR4-mediated TRIF-dependent pathway focusing on TRIF, TRAM, IRF-3, and IFN-β; and 2) the MyD88-dependent pathway focusing on MyD88, NF-κB and IL-1β. The results showed that TRAM, IRF-3 and IFN-β were down-regulated by DGLHD partially, but as a whole, DGLHD treatment did not significantly affect the expression of these two additional signal molecules in pancreas (Fig. 11B). These results indicate that DGLHD appears to exert its anti-inflammatory and antidiabetic actions at least in part by inhibiting the JAK2-STAT3 pathway, but not by affecting either TLR4-mediated TRIF-dependent or MyD88-dependent pathways.

### DGLHD decreased JAK2, STAT3 protein expression and increased SOCS3 level in pancreas, spleen, thymus and DCs

**Figure 12:**
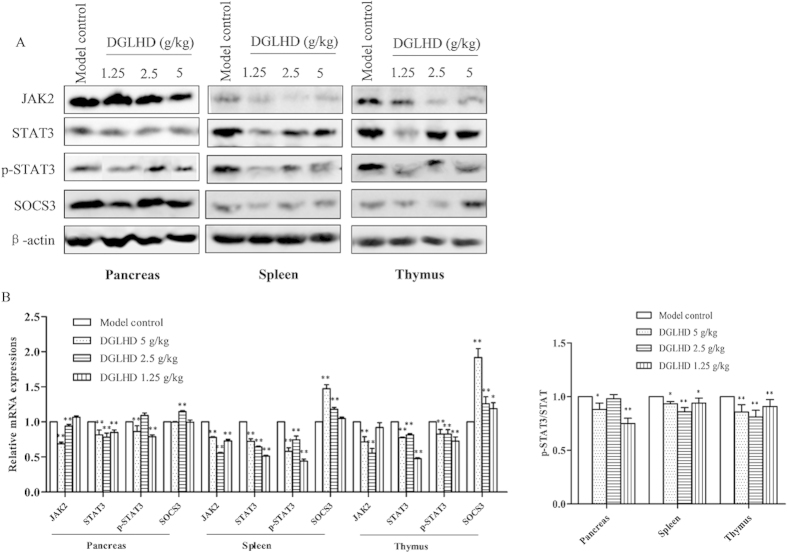
DGLHD decreased JAK2, STAT3 protein expression and increased SOCS3 level in the pancreas, spleen and thymus. (**A**) Respectively western blot results showed the expressions of JAK2, STAT3, p-STAT3 and SOCS3 in 12 week-old NOD mice treated with DGLHD for 4 weeks from 8 until 12 weeks and model control group. β-actin was used as loading control. (**B**) Bar graphs showed semi-quantitative evaluation of their expression by densitometry. Data were presented as means ± SD (*n *= 3). ^*^P < 0.05, ^**^P < 0.01 compared with model control group.

To confirm the above results, we measured JAK2, STAT3, and SOCS3 protein levels. The results showed that DGLHD down-regulated the expressions of JAK2, STAT3 and decreased the phosphorylation of STAT3, while up-regulating the inhibitor protein SOCS3 significantly (Fig. 12A,B). The results strongly suggest that inhibiting JAK2-STAT3-dependent signaling pathways in various tissues by DGLHD is involved in its therapeutic actions in this model of NOD.

## Discussion

T1DM is an autoimmune disease characterized by inflammatory cell infiltration and the destruction of pancreatic islet β cells^22^. Conventional drugs for T1DM mainly depend on exogenous insulin supplement and promoting insulin secretion by islet β cells. But with the development of the disease, β cells are damaged seriously, the treatment will not be adequate. Newer therapies designed to protect and repair islet β cells or to preserve immune homeostasis will be two critical strategies for T1DM treatment in the future^23^.

In the study reported here, we focused on a Chinese traditional therapy, DGLHD, and its mechanisms of action in improving pathophysiologic characteristics of diabetes in NOD mice. The results showed that treatment with DGLHD: 1) increased insulin secretion and sensitivity and reduced the incidence of diabetes (Fig. 1); 2) decreased the extent of pancreatic β cell damage (Fig. 4); 3) decreased the extent of islet inflammatory responses (Fig. 2); 4) down-regulated the production of Th1-type cytokines (IFN-γ and IL-2) and up-regulated the production of Th2-type cytokines (IL-10 and TGF-β_1_) (Fig. 3) during the progression of diabetes. In the experiments, we found that the efficacy of DGLHD at low dose was better in some indicators; higher doses, though somewhat less effective, seemed to be free of serious adverse effects. Dose-effect relationships of prescriptions and herbal medicines of this type are often complicated by mixtures of active ingredients, multiple therapeutic targets, and different detecting indicators^24^.

In T1DM, progressive islet β cell failure and apoptosis is induced by lymphocyte infiltration^25^. AAT-1 is a liver-derived multifunctional serpin with anti-inflammatory and anti-apoptotic properties for islet β cell^13^. Here we showed that DGLHD elicited higher levels of AAT-1 in serum and DCs supernatants from NOD mice (Fig. 4A), implicating endogenous AAT-1 in protective effects of DGLHD on islet β cells. We also found that DGLHD and active ingredients increased the expressions of the anti-apoptotic gene Bcl-2 and the proliferation-related gene CyclinD1, decreased the expression of the pro-apoptosis gene Bax, and increased the ratio of Bcl-2/Bax in NIT-1 cells, a model murine pancreatic β cell line. All of these observations are consistent with islet β cell protecting and repairing functions of DGLHD in this model of T1DM.

Both cellular immunity and nonspecific immunity play important roles in the onset and development of T1DM. DCs, T lymphocytes, B lymphocytes, and macrophages, among other cell types, participate in the immune regulation of T1DM^26^. Regulating the differentiation of immune cells is a potential new strategy for the treatment T1DM^27^. Specifically, CD4^+^CD25^+^Foxp3^+^ T_regs_ are important in the maintenance of immunological tolerance^28^. T_regs_ mediate immune suppressive effects by several mechanisms, including anti-inflammatory cytokine induction, cytotoxic effects, metabolic disruption, and DCs regulation^29^. The appropriate balance between effector T cells and T_regs_ is necessary for self-tolerance and normal immune responses *in vivo*. Our experiments showed that DGLHD, its principal component ferulic acid, and adjuvant components baicalin and berberine suppressed T lymphocyte proliferation (Fig. 5). We also found that DGLHD increased T_regs_ proportion and Foxp3 expression in pancreas, spleen, and thymus of NOD mice (Fig. 6).

On the other hand, DCs are professional APCs that are critical for induction of both adaptive immunity and tolerance. Disorder of DCs leads to the presentation of auto-antigens followed by the activation of reactive T cells. DCs exist in mature (mDCs) and immature (iDCs) states. iDCs express low levels of MHC-I and co-stimulation CD80/CD86/CD40 molecules, and induce immune tolerance, promoting the conversion of T cells into CD4^+^CD25^+^Foxp3^+^ T_regs_. mDCs can activate the immune response, induce T cell differentiation into Th cells^30^. Our studies found that DGLHD down-regulated the expression of CD86 and MHC-II and enhanced the dextran-uptake ability of DCs (Fig. 7). DGLHD also decreased the prevalence of the chemokine receptor CCR7 on DCs, and inhibited CD11c^+^CFSE^+^ DCs migration to mesenteric LN and inguinal LN. The results showed that DGLHD reduced the migration and increased the antigen uptake of DCs, which induced the immune tolerance.

Unconventional DCs subset, termed mcDCs, can reverse T cell anergy. The effectiveness of mcDCs at diminishing T cell tolerance had been demonstrated in the context of tumors and autoimmunity^31^. Our studies found that DGLHD impeded mcDCs differentiation in diabetic mice in LN, but not in spleen, BM, or thymus (Fig. 9). The results indicated that DGLHD might modulate mcDCs in LN to maintain T cell tolerance.

PD-L1 is an interesting molecule that is uniquely involved in regulation of the immune response. It is a key co-inhibitory molecule expressed on DCs^32^, and is central in inducing and maintaining immune tolerance. Interactions between PD-L1, expressed on DCs, and PD-1, on T cells, suppress T cells immune activities^33^. Our studies showed that DGLHD increased the expression of PD-L1 on DCs (Fig. 10A), which inhibited the ability of DCs to stimulate T lymphocyte proliferation (Fig. 10B,C) and promoted the actions of DCs to induce T cell tolerance (Fig. 6). Thus, in this model of T1DM, DGLHD treatment strongly influenced both the phenotype and function of DCs, with favorable consequences on T cell tolerance and T lymphocyte proliferation.

The JAK2-STAT3 is an important signal transduction pathway mediating a variety of cellular pathophysiologic processes, including inflammatory responses, oxidative stress, cell damage, and apoptosis^34^. SOCS, a suppressor of cytokine signaling protein, is an important physiological inhibitor to the JAK2-STAT3 pathway^35^. Recent studies implicated SOCS3 in innate immune cell function, acquired immune and inflammatory responses, Th1/Th2 immune imbalances, and, notably, insulin signal transduction^36^. Our studies found that DGLHD down-regulated the expression of JAK2, STAT3, and the phosphorylation of STAT3, and up-regulated the expression of SOCS3 (Figs 11 and 12). These results are consistent with the hypothesis that palliative action of DGLHD in T1DM involves modulation of JAK2-STAT3 pathways and possibly SOCS3 function.

In TCM, combinatory therapeutic strategies based on patient symptoms and characteristics, are often prescribed to treat diabetes and other diseases^9^. Advantages of formulae composed of a mixture of natural products include the presumed ability to target multiple sites. DGLHD, for example, has been well established recently as an effective treatment of T1DM^37^. Modern medical studies have shown that *Angelica sinensis* and the main ingredients from *radix rehmanniae* have the hypoglycemic and hypolipidemic effects^38^. In this study, we found that DGLHD and its active ingredients (ferulic acid, catalpol, baicalin, berberine, and astragaloside IV) increased glucose uptake in HepG2 cells and insulin secretion in NIT-1 cell line. They also promoted the proliferation and inhibited the apoptosis of NIT-1 cell line. The individual ingredients, to different degrees, also decreased T lymphocyte proliferation and DC activity of stimulating T cell proliferation *in vitro*. The combination was more effective than any individual ingredient but, notably, was not associated with any apparent increase in the incidence or severity of adverse effects. Our study thus provides the first direct evidence in support of the rationale for treatment of T1DM by a combination of multiple natural products in one formula.

## Materials and Methods

### Drugs

DGLHD was provided by the Dept. of Traditional Chinese Medicine, the Central Hospital of Wuhan (batch number: 20140910). The formula, including *angelica sinensis*, *radix rehmanniae*, *radix rehmanniae preparata*, *radix scutellariae*, *rhizoma coptidis*, *cortex phellodendri* and *radix astragali* at the ratio of 1:1:1:1:1:1:2, was soaked in distilled water and concentrated to a final density of 0.13 g/ml. Quality control was carried out by high performance liquid chromatography (HPLC) according to the method described in “Chinese Pharmacopoeia” (data not shown). The five active ingredients, ferulic acid (batch number: 110773–201313), catalpol (batch number: 2415-24-9), baicalin (batch number: 21967-41-9), berberine (batch number: 2086-83-1) and astragaloside IV (batch number: GZDD-0093) were bought from the National Institutes of Food and Drug Control (Beijing, China).

### Mice

Animal experiments were approved by *the Institutional Animal Care and Use Committee of Tongji Medical College, Huazhong University of Science and Technology*. Animal care and experimental procedures were carried out in accordance with *the guidelines of the Institutional Animal Care and Use Committee of Tongji Medical College* and *the National Institutes of Health Guide for the Care and Use of Laboratory Animals.* Female, 6 week-old NOD mice were purchased from Beijing HFK BioTechnology Co. Ltd. Experimental colonies were maintained at the experimental animal center of Tongji Medical College (Huazhong University of Science and Technology, China) under specific pathogen-free conditions. The animals were kept in cages at 23 ± 2 °C and fed with standard laboratory diet and tap water throughout the experiments.

### Cell cultures

(1) Preparation of DCs and T lymphocytes^39^.

Bone marrow (BM) cells or spleen lymphocytes were collected from the tibia and femur or spleen of NOD mice. The cells were cultured in RPMI-1640 medium containing 10% fetal bovine serum (FBS) at a density of 5 × 10^6^ cells/ml in 6-well plates or 5 × 10^5^ cells/ml in 96-well plates. DCs were cultured with cytokines GM-CSF (20 ng/ml) and IL-4 (10 ng/ml), and T cells with ConA (10 μg/ml). In addition, we defined these DCs with CD11c^+^ using flow cytometry (FACS).

(2) HepG2 and NIT-1 cell lines.

HepG2 is a human liver cancer cell line obtained from Shanghai Institute of Cell Biology, Chinese Academy of Sciences. NIT-1 is a murine pancreatic β cell line obtained from Tongji Medical College. HepG2 was grown in a high glucose DMEM medium and NIT-1 in a low glucose DMEM medium, respectively, in a humidified atmosphere containing 5% CO_2_ at 37 °C. Cells in the exponential growth phase were used in the experiments.

### Reagents and kits

Mouse interferon (IFN)-γ, interleukin (IL)-2, IL-10, transforming growth factor (TGF)-β_1_, α_1_-antitrypsin (AAT-1), insulin (INS) enzyme-linked immunosorbent assay (ELISA) kits, and mouse regulatory T cell staining kit were purchased from eBioscience, USA. Antibodies used for FACS analysis were purchased from BD Pharmingen, USA, total RNA isolation system and reverse transcription (RT) system from Promega, Madison, WI, RPMI-1640 and FBS from Gibco, USA, 3-(4,5-dimethylthiazolyl)-2,5-diphenyltetrazolium bromide (MTT) from Sigma, St. Louis, MO, ECL chemiluminescence detection kit from Amersham Biosciences UK Ltd., Buckinghamshire, UK, and glucose test kit from Rsbio, Shanghai, China.

### Groups and treatments

8 week-old NOD mice were randomly divided into four groups as follows: model control group (orally administered saline, five days a week for 4 weeks), 5, 2.5 and 1.25 g/kg DGLHD groups (orally administered DGLHD at different concentrations five days a week for 4 weeks). After the 4-week treatment period, 6 mice at 12-weeks of age per group were randomly selected to be sacrificed to perform oral glucose tolerance tests (OGTT), insulin tolerance tests (ITT), insulin secretion tests and histopathological analysis of insulitis. Mice from these groups were also used to analyze spleen T lymphocyte proliferation, the percentage of CD4^+^CD25^+^Foxp3^+^ T_regs_, DCs phenotype and function, and possible signaling pathways activated by DGLHD treatment. Blood glucose levels were monitored weekly until the mice were 26 weeks of age to assess the incidence and severity of diabetes. *In vitro*, experiments were divided into 7 groups: controls, DGLHD (10 μg/ml), and treatment groups for five active ingredients (ferulic acid, catalpol, baicalin, berberine, and astragaloside IV, 10 μg/ml) groups. DGLHD or its five active ingredients were co-cultured with DCs, T lymphocytes, HepG2 cell line and NIT-1 cell line for 48 h, respectively.

### Assessment of diabetes

The development of diabetes was monitored by measuring tail vein blood glucose twice weekly using the ACCU-CHEK III system (Roche Diagnostics Ltd., Shanghai, China). A mouse with a non-fasted blood glucose level above 16.7 mM for 2 consecutive weeks was considered diabetic.

### OGTT and ITT tests

After overnight fasting, mice were orally administered 2 g/kg glucose for OGTT. For ITT, after 5 h of fasting, mice were injected intraperitoneally with 0.75 U/kg insulin. Blood glucose was measured at 0, 15, 30, 60, 90, and 120 min after orally administered glucose or injected intraperitoneally with insulin.

### HepG2 glucose uptake test

HepG2 cells were cultured with DGLHD or its different five active ingredients for 48 h after starving cultured for 24 h. The concentration of glucose in the medium was tested by the glucose oxidase-peroxidase method as manufacturer’s protocol.

### Histology and grading of islet infiltrates

Pancreata from mice were embedded in paraffin. Insulitis was graded in at least 10 islets per pancreas after hematoxylin-eosin (HE) staining. The degree of insulitis was graded according to the following: normal islet, score 1; perivascular/periductal infiltration, score 2; peri-insulitis, score 3; mild insulitis (<25% of the islet infiltrated), score 4; and severe insulitis (more than 25% of the islet infiltrated), score 5^40^. The number of islets was counted and represented as islet number per square centimeter. The mean insulitis score for each pancreas was calculated by dividing the sum of graded islets by the total number of islets analyzed.

### ELISA

Serum and supernatant samples of the mice, DCs and NIT-1 cell line with or without DGLHD treatment were collected. The concentrations of Th1 cytokines IL-2, IFN-γ; Th2 cytokines IL-10, TGF-β_1_, pancreas islet β cell repair cytokine AAT-1, and insulin secretion level were determined by quantitative sandwich ELISA using ELISA kits.

### Spleen T lymphocyte proliferation^41^

For testing the effects of DGLHD and its five active ingredients on Con A-induced T lymphocyte proliferation *in vivo* and *in vitro*, the spleens were ground and passed through nylon meshes and cells were isolated by gradient centrifugation with Ficoll solution. The proliferation of T lymphocyte was investigated by MTT assay at the absorbance of 490 nm.

### Flow cytometry (FACS) analysis for the phenotype of BMDCs and the percentage of merocytic dendritic cell (mcDCs) and T_regs_

The expression of surface molecules and intracellular staining were analyzed using a FACS scan (Becton Dickinson, Mountain View, CA) as described previously^42^. BMDCs were sustained at 4 °C for 30 min with the following antibodies diluted in PBS: APC-CD11c; FITC-CD86; PE-MHC-II. The percentage of CD4^+^CD25^+^Foxp3^+^ T_regs_ in splenic lymphocytes was incubated with FITC-CD4, PE-CD25 and intracellular staining of PE-Cy5-Foxp3. Non-diabetic NOD and pathogenic NOD mice, with or without DGLHD treatment, were used to evaluate the expression of mcDCs. The proportion of mcDCs was sustained with PE-Cy7-CD11c; APC-CD11b; FITC-CD8a; PE-PDCA.

### FITC-conjugated dextran uptake

Quantitative analysis of FITC-conjugated dextran uptake by DCs was performed as described by Huggins A *et al*^43^. BMDCs (2 × 10^6^ /ml) were incubated in complete medium with FITC-conjugated dextran 1 mg/ml at 37 °C for 60 min. Thereafter, cells were washed 2 times with PBS, stained with APC-CD11c, and analyzed by FACS. CD11c^+^ populations were gated, and the mean fluorescence intensity of the FITC cells was calculated.

### The migration of DCs

BMDCs (2 × 10^6^ /ml) isolated from NOD mice, untreated or treated with DGLHD at 5, 2.5, or 1.25 g/kg, were cultured as above. Sustained with PE-Cy7-CD11c and APC-CCR7, the migration ability of DCs was calculated using the percentage of CD11c^+^/CCR7^+^ gated cells. For subsequent studies, BMDCs isolated from NOD mice treated with or without DGLHD were labeled for 20 min with CFSE. The DCs were resuspended in 50 μl of PBS and injected into the hind paw of normal mice subcutaneously. After 48 h, the mesenteric lymph node (LN) and inguinal LN were harvested and processed into a single-cell suspension stained with APC-CD11c, and analyzed by FACS. The number of migrated DCs was calculated using the percentage of CD11c^+^/CFSE^+^ gated cells^43^.

### Mixed lymphocyte reaction (MLR)

DCs isolated from NOD mice treated by DGLHD *in vivo* or normal DCs treated by DGLHD and its active ingredients for 48 h *in vitro* were cultured as above. T lymphocytes and DCs were co-cultured at a ratio of 5:1, 10:1, or 20:1 for 48 h. The proliferation of T lymphocytes was measured using MTT assays, and the absorbance at 490 nm was determined using a Multi-Well Plate Reader.

### Quantitative real time PCR (qPCR) analysis

RNA was isolated from pancreas, spleen, thymus, and BMDCs using TRIzol reagent according to the manufacturer’s protocol. Total RNA was reverse transcribed into cDNA using a transcription kit. The resulting cDNA was amplified by denaturation at 95 °C for 5 min, 40 cycles of denaturation at 95 °C for 10 s, annealing at 55–60 °C for 20 s, and extension at 72 °C for 30 s. The corresponding primer sequences were listed in Table 2.

### Western blotting analysis

Proteins were separated by SDS-PAGE and transferred onto a polyvinylidene difluoride (PVDF) membrane by electro-blotting. Membranes were incubated overnight at 4 °C with various mouse monoclonal antibodies at 1:1000 dilution. After incubation with the secondary antibody, proteins were detected with an ECL chemiluminescence detection kit. The amount of protein expression was corrected by the amount of β-actin in the same sample.

### Statistical analysis

Statistical analyses were performed using the SPSS 11.0 software program (SPSS Software Products, Chicago, IL, USA). All data in this study were presented as means ± standard deviation (SD). Statistical analysis among groups was performed by one-way analysis of variance (ANOVA). Statistical analysis between two groups was performed using Student’s *t*-test. Results were considered statistically significant at *P *< 0.05.

## Additional Information

**How to cite this article**: Liu, T. *et al.* Interaction of dendritic cells and T lymphocytes for the therapeutic effect of *Dangguiliuhuang* decoction to autoimmune diabetes. *Sci. Rep.* **5**, 13982; doi: 10.1038/srep13982 (2015).

## Figures and Tables

**Table 1:** Biological activities of individual herbs in the DGLHD.

		**Islet β cell protection**	**Hypoglycemic**	**Hypolipidemic**	**Insulin sensitivity**	**Diabetic complications**
	DGLHD	+	+	+	+	+
Principal components	*Angelica sinensis*		+	+		
	*Radix rehmanniae*		+			+
	*Radix rehmanniae preparata*		+			+
Adjuvant components	*Radix scutellariae*			+		+
	*Rhizoma coptidis*		+	+	+	
	*Cortex phellodendri*		+	+	+	
	*Radix astragali*	+	+			+

**Table 2:** PCR Primers used in study.

**Gene**	**Sense**	**Antisense**
Bcl-2	GGC TAC GAG TGG GAT GCT	CCA CCG AAC TCA AAG AAG G
Bax	AAG CTG AGC GAG TGT CTC AAG	CAA AGT AGA AAA AGG GCG ACA AC
CyclinD1	GCA GGT CTG TGA GGA GCA	AGC ATA AAG AGG GAT TGT CG
Foxp3	GAC ATC CCA TAT TCT CCC A	GAC GTG AAG CCT AGA CAG C
PD-L1	CAA AGA ATT TTG GTT GTG GA	AGC TTC TCC TCT CTC TTG GA
JAK2	TTC CTC CAT AGC AAA CAG	GTG AGA AGT GAA GGT CCA G
STAT3	TGG GTT TCA TCA GCA AGG	AGG TGG AGG AAG TGA TAC AGG AGG C
TLR4	CGC TTT CAC CTC TGC CTT CAC TAC AG	ACA CTA CCA CAA TAA CCT TCC GGC TC
MyD88	AGC AGA ACC AGG AGT CCG AGA AGC	GGG GCA GTA GCA GAT AAA GGC ATC G
IL-1β	CGC AGC AGC ACA TCA ACA AGA GC	TGT CCT CAT CCT GGA AGG TCC ACG
NF-κB	GCT TTG CAA ACC TGG GAA TA	TCC GCC TTC TGC TTG TAG AT
TRIF	ATG GAT AAC CCA GGG CCT T	TTC TGG TCA CTG CAG GGG AT
IFN-β	TTC CTG CTG TGC TTC TCC AC	GAT TCA CTA CCA GTC CCA GAG TC
TRAM	ATG GCC AGT CCT GGA CTT C	CAA GCA GGC TTC CTC AGA ATT
IRF-3	AAC AAT GGG AGT TCG AGG TG	TCC TTG TGG ACC TCT CCA TC
β-actin	TGC TGT CCC TGT ATG CCT CT	TTT GAT GTC ACG CAC GAT TT
